# *Mycobacteria* as evolutionary drivers of host innate immunity: insights from comparing experimental host models

**DOI:** 10.3389/ftubr.2025.1735950

**Published:** 2025-12-12

**Authors:** Mariona Cortacans, Pere-Joan Cardona

**Affiliations:** 1 Experimental Tuberculosis Unit (UTE), Institut de Recerca Germans Trias i Pujol (IGTP), Badalona, Spain; 2 Microbiology and Genetics Department, Universitat Autònoma de Barcelona, Bellaterra, Spain; 3 Servei de Microbiologia, Laboratori Clínic de la Metropolitana Nord (LCMN), Hospital Universitari Germans Trias i Pujol (HUGTiP), Badalona, Spain; 4 Centre de Medicina Comparativa i Bioimatge de Catalunya (CMCiB), Badalona, Spain; 5 Centro de Investigación Biomédica en Red de Enfermedades Respiratorias (CIBERES), Madrid, Spain

**Keywords:** mycobacteria, evolution, virulence, tuberculosis, innate immunity, non-mammalian models

## Abstract

The genus *Mycobacterium* exerts a strong selective force, shaping the evolution and structure of innate immune systems across various hosts and revealing overarching, conserved principles of host defense. Despite their phylogenetic distance, amoebae, nematodes, insects, wax moth larvae, and zebrafish share fundamental innate immune strategies while also exhibiting key differences in tissue organization, immune complexity, and the presence or absence of adaptive immunity. This comparative review synthesizes insights from these systems to highlight both the conserved mechanisms that mycobacteria repeatedly exploit and the lineage-specific features that shape host susceptibility. Amoebae demonstrate ancient, cell-autonomous defenses, including nutritional immunity through metal trafficking (Nramp1/zinc intoxication) and membrane repair pathways (ESCRT/autophagy) against the ESX-1 system. Moving to metazoans, the importance of conserved signaling, such as the p38 MAPK (PMK-1) pathway in *C. elegans*, becomes evident, which *M. marinum* actively suppresses via VHP-1. In other invertebrates, such as *Drosophila*, integrated immunometabolism is present, in which disruption of the Akt–FOXO axis causes a conserved wasting syndrome, and *Galleria* mimics chronic TB pathology by forming granuloma-like structures with lipid-accumulating hemocytes and demonstrating innate immune priming. Larval zebrafish, which depend solely on innate immunity, show pathogen-driven granuloma formation and spread, with ESX-1 mediating pro-necrotic cell death and the Asc-dependent inflammasome contributing to restriction. Overall, these cross-species comparisons demonstrate how mycobacteria exploit foundational host mechanisms while revealing the evolutionary breadth and limits of innate immune strategies across the animal kingdom.

## 1 Introduction

Historically, the study of mycobacteria has primarily focused on clinical and molecular views of *Mycobacterium tuberculosis* (Mtb) and its interactions with humans (1–9). However, considering the evolutionary and ecological background in which these bacteria developed shows that mycobacteria are not just pathogens, but also powerful selective forces that have influenced the innate immune systems of many different organisms over evolutionary time.

**Figure 1:**
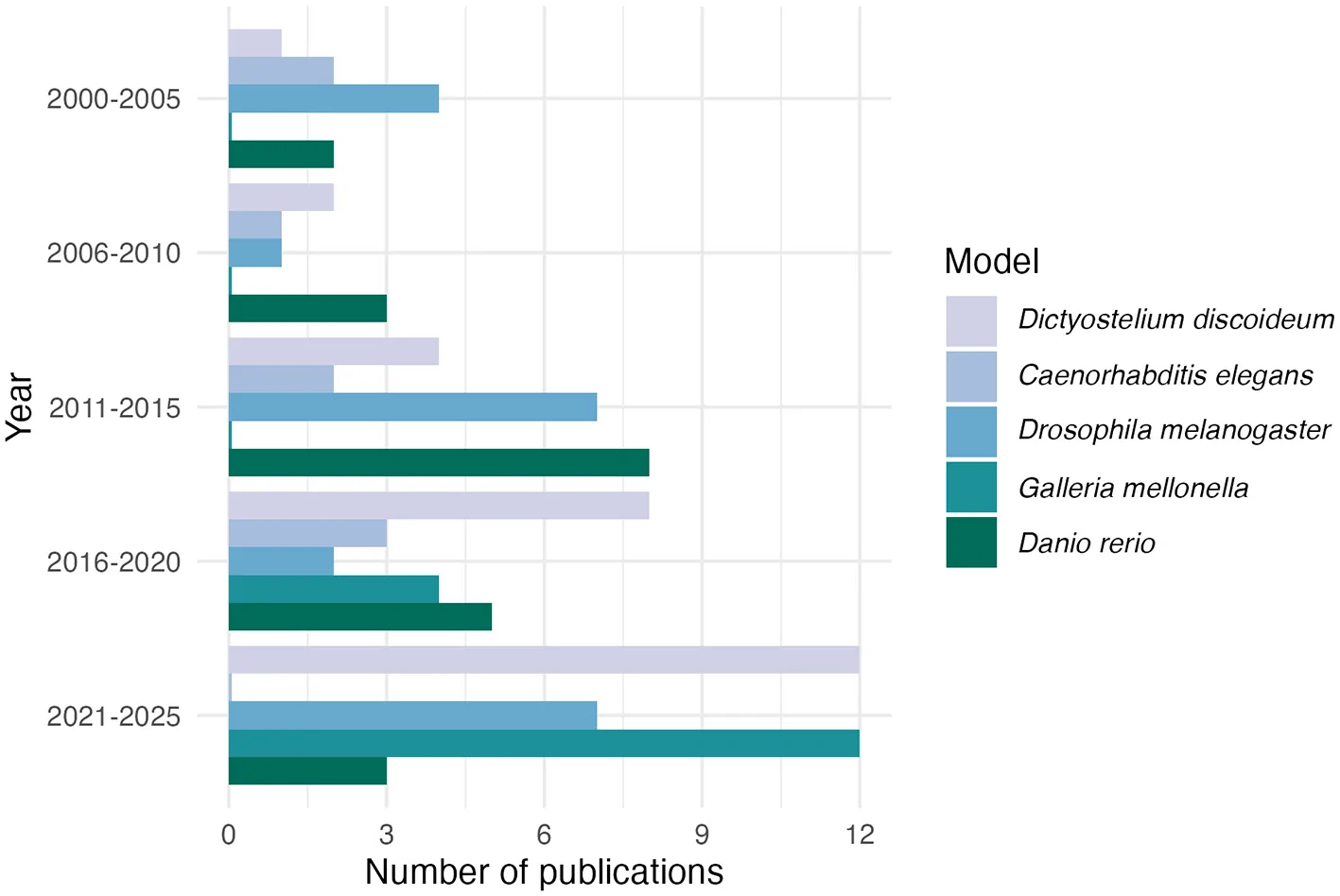
Trends in the use of non-mammalian models to study innate immunity during mycobacterial infection (2000–2025). The bar chart shows the number of PubMed-indexed original research articles employing each model organism across 5-year intervals. Research using *Danio rerio* (zebrafish) has increased steadily since 2010, reflecting its prominence as a vertebrate model for granulomatous infection. *Dictyostelium discoideum* and *Drosophila melanogaster* remain widely used, whereas studies in *Caenorhabditis elegans* and *Galleria mellonella* have expanded more recently to explore epithelial and humoral aspects of innate immunity. Search strategy used: *(mycobacterium) AND (“danio rerio”) AND (innate immunity OR macrophage OR inflammation OR inflammatory response OR autophagy OR xenophagy) NOT review [Publication Type]*.

Their ability to survive within phagocytic cells, endure stress, and manipulate host responses reflects adaptations that likely originated long before the emergence of vertebrates (3, 4, 10–15). Understanding these interactions through comparative host models provides unique insights into how ancient host–microbe conflicts shaped the cellular and molecular foundations of immunity. Modern research now employs a wide range of host models to explore these interactions. In fact, publication trends show a significant increase in original research using non-mammalian hosts to dissect innate immune responses to mycobacterial infection (Figure 1). The past 20 years have shown a steady diversification of experimental systems—from *Dictyostelium discoideum* and *Caenorhabditis elegans* to *Drosophila melanogaster, Galleria mellonella*, and *Danio rerio*—highlighting a growing recognition of their ability to reveal conserved immune principles. This growth underscores a shift from solely mammalian perspectives to an integrated, evolutionary approach to studying host–pathogen interactions.

The fitness of any organism depends on its environment, and for pathogens, the host is a crucial part of that environment. Understanding pathogen evolution therefore requires examining their interactions with host cells. In early eukaryotes, phagocytic pathways originally evolved for bacterial predation, and only later did coevolution with bacteria that acquired resistance and, eventually, virulence traits drive the refinement of immune defenses. These interactions are therefore the main drivers of host-pathogen coevolution.

This review combines insights from five key experimental systems (amoebae, nematodes, insects, wax moth larvae, and zebrafish) to show how mycobacteria act as evolutionary forces shaping innate immunity. A comparative overview of these models is provided in Table 1. Instead of examining pathogenesis alone, we highlight reciprocal adaptation: how host defenses have evolved in response to ongoing mycobacterial challenges, and how bacterial survival tactics reveal ancient principles of immune organization.

**Table 1:** Comparative overview of non-mammalian and early vertebrate models used to study mycobacterial innate immunity.

**Model**	**Temp (°C)**	**Principal innate components**	**Relevant mycobacterial species and infection outcome**	**Conserved host pathways & effectors**	**Mycobacterial strategies revealed**	**Key evolutionary insights**	**Strengths**	**Limitations**	**References**
Amoebae	22–27 (Dd) 30–37 (Ac)	- Phagocytic vacuoles/lysosome fusion - Divalent cation transporter Nramp1 (Slc11a1 homolog) - ESCRT and autophagy machinery - LRR and NLRs - DNA ETs produced by Sentinel cells	- *M. marinum, M. avium, M. bovis, M. abscessus*- Outcomes range from killing to stable intracellular residence (AROs) - Cysts (and spores in social amoebae) protect bacteria enabling environmental persistence	- Nramp1-mediated metal export - Zn^2+^ intoxication - LmpA/LmpB (LIMP-2/CD36-like) for phagosomal acidification - ESCRT and autophagy (TrafE-orchestrated)	- ESX-1/EsxA/PDIM damage of the MCV leading to cytosolic access - TORC1-dependent autophagy flux inhibition - Ejectosome-mediated ejection (actin-dependent, non-lytic) for exit and spread - Upregulation of efflux transporter CtpC	- “Proto-macrophage” paradigm: evolutionary training ground for intracellular pathogens - Manipulation of metal availability - Shared virulence strategies suggest co-evolution in environmental niches - DNA ET production evolved before metazoans - Multicellular stages with Sentinel-cell DNA ETs	- Combines unicellular simplicity with conserved phagocyte machinery - Powerful cell biology and genetics - Live dissection of phagosome–pathogen conflict - Temp overlap with human pathogens (Ac)	- Lacks p38 MAPK homolog - Lacks canonical TLRs/cytokine networks - Simplified insulin axis (no insulin/IGF ligands) - Lacks canonical inflammasome and pyroptotic pathways	(14, 41, 47–49, 54–61, 64, 66, 70–73, 75–81)
*Caenorhabditis elegans*	20–25	- No professional phagocytes - Intestine (20 non-renewable cells) is barrier/immune organ - Core PMK-1/p38 MAPK, SKN-1 pathways (Nrf1/2) - Metal homeostasis pathways	- *M. marinum*: >80% mortality/24 h; pathology (depigmentation, bagging) - *M. smegmatis*: < 15% mortality	- PMK-1 controls C-type lectins/CUB proteins - SKN-1 limits self-ROS damage - Conserved autophagy (*atg1-10*) - ESCRT-mediated membrane repair	- VHP-1 phosphatase suppresses PMK-1 - Gut persistenc, villus disruption - Probable actin manipulation reminiscent of macrophages	- Bridges unicellular → multicellular defenses - Epithelial, cell-autonomous programs informative for macrophage biology	- Genetic clarity - Stark pathogenic vs. non-pathogenic readouts	- Lacks NF-κB/IKK/Toll → MyD88 and canonical Toll signaling cascades - No phagocytes - MTBC temp constraints - Mycobacteria are not natural pathogens	(95–97, 99, 106–111, 116–123)
*Drosophila melanogaster*	25–29	- Hemocytes (plasmatocytes) = MΦ - Thanacytes (cytotoxic) - Class B scavenger receptor (Pes) - Toll/IMD → NF-κB (Dorsal/Dif, Relish) - JAK/STAT signaling - NAIP-like/CED-4 sensors - p38 MAPK pathway	- *M. marinum*: grows in hemocytes leading to cachexia - *M. abscessus*: highly virulent, resists hemocyte cytotoxicity - *M. smegmatis*: restricted	- Phagocytosis by hemocytes - Pes (CD36/SR-B-like) uptake - Akt–FOXO signaling cascade drives wasting - MEF2 switches coupling anabolism and immunity - ESCRT-III components	- Blocks phagosome acidification - Survives after hemocyte apoptosis - Shields m-DAP (weak/late IMD), paradoxical Toll activation later - Manipulates host Akt-FOXO - Represses Atg2 via JAK/STAT (Upd2/Upd3) to promote lipid accumulation	- High degree of conservation in disease causing genes - Dissects conserved immune–metabolic trade-offs (cachexia-like wasting), autophagy–lipid crosstalk, and macrophage-analog evasion	- Genetics + *in vivo* + S2 screens - Cellular & humoral arms of innate immunity	- Often surrogate (*M. marinum* vs. Mtb) - Weak/late AMP to *M. marinum*- No true inflammasome - MTBC temp constraints - Mycobacteria are not natural pathogens	(125–129, 132–136, 139, 140, 142–145, 148)
*Galleria mellonella*	37	- Hemocytes = MΦ/“neutrophil-like” - Humoral AMPs, melanisation (complement-like), hemolin opsonin, ROS/RNS - p38 MAPK pathway - Autophagy (Atg8)	- Supports Mtb H37Rv, *M. bovis* BCG, and NTM (i.e., *M. abscessus, M. marinum, M. fortuitum*) - GLS can fail for H37Rv leading to dissemination & death	- Phagocytosis by hemocytes - Cecropins, gloverins (α-helical, LL-37-like) - Formation of GLS/nodules - TAG-rich foamy hemocytes mirror human granulomas	- Intracellular growth in hemocytes - *M. abscessus* suppresses melanisation and co-infection immunity - Virulence lipids PDIM and PGL involved in pathogenesis	- Models granuloma-like sequestration - Mimics lipid accumulation seen in foamy macrophages - Dormancy-like metabolic niches at 37 °C - Evidence of immune priming	- MTBC at human-relevant temperature - Virulence ranking - Useful for studying chronic TB	- Fewer genetic tools - GLS are only vertebrate granuloma analogs - Constraints in having to work with larvae - Mycobacteria are not natural pathogens	(150–157, 160, 162–167)
*Danio rerio*	28.5–33	- MΦ and neutrophils ~1 dpf - Asc-dependent inflammasome - Autophagy machinery (Dram1, Optn, p62) - TNF pathway - TLR signaling (MyD88)	- Natural host for *M. marinum*- Recreates organized, caseating granulomas and core TB hallmarks	- TLR–MyD88 pathway - Asc-inflammasome - ROS/NO production via NADPH oxidase - Hif-1 signaling modulating NO - CXCR3–CXCL11 chemotaxis	- ESX-1 drives macrophage necrosis/pyroptosis - ESAT-6 induces MMP-9 (macrophage chemotaxis) - MarP enables acid tolerance in phagolysosomes - PDIM and PGL involved in pathogenesis	- Visualizes pathogen-driven granuloma biogenesis, macrophage dissemination, and inflammatory balance - Highlights the role of ESX-1 in driving granuloma pathology - Confirms the requirement for a balanced inflammatory response	- Vertebrate context - Live granuloma imaging - Stage-specific innate vs. adaptive immunity - *M. marinum* is a natural pathogen	- Often surrogate (*M. marinum* vs. Mtb) - Some inflammatory phenotypes are model-specific - MTBC temperature constraints	(66, 172, 173, 175, 176, 178, 180, 182, 185, 186, 188, 189, 191–200)

The table summarizes key biological features, immune components, relevant mycobacterial species, conserved host pathways, and principal evolutionary insights derived from each model system discussed in this review. Together, these models illustrate the progressive emergence of conserved innate immune mechanisms and host adaptations to intracellular mycobacterial infection. Strengths and limitations highlight each model's suitability for dissecting specific aspects of host–pathogen interactions and evolutionary conservation.

## 2 Mycobacteria at the crossroads of ecology, pathogenicity, and host evolution

### 2.1 Diversity and ecological breadth of the genus

The genus *Mycobacterium* is a highly diverse taxonomic entity, encompassing 170–200 recognized species, whose broad genetic content is reflected in its open pan-genome (16–19). This diversity spans a wide array of ecological lifestyles, beginning with organisms commonly found in environmental reservoirs such as water and soil, from which the *M. tuberculosis* complex (MTBC) evolved (3, 16, 17). At the end of the pathogenic spectrum are obligate pathogens, primarily the MTBC, which includes Mtb (5, 17) and *Mycobacterium leprae* (16). The remainder of the genus, referred to as non-tuberculous mycobacteria (NTM), consists mainly of environmental organisms that often exhibit opportunistic pathogenicity and can cause severe diseases such as pulmonary infections, lymphadenitis, or disseminated disease, particularly in immunocompromised individuals or those with pre-existing lung conditions (16, 19). Some of these organisms include the fast-growers *Mycobacterium abscessus* and *Mycobacterium fortuitum*; and the slow-growers *Mycobacterium ulcerans, Mycobacterium avium, Mycobacterium marinum, Mycobacterium xenopi, Mycobacterium gordonae*, and *Mycobacterium kansasii* (3, 19), among many others.

The ability of mycobacteria to establish persistence in hosts is central to their evolutionary success, although recent evidence indicates that the traditional estimate of MTBC latency in one-third of the global population is a substantial overestimate, with only a small minority of immunoreactive individuals (likely between 1% and 11%) harboring viable latent bacteria (8, 20, 21). Such long-term persistence underscores an intimate host–pathogen coevolution that has unfolded over millennia, driving bacterial adaptations finely tuned to the intracellular environment of macrophages. Genomic and functional analyses highlight key virulence systems that allow mycobacteria to resist immune clearance, modulate host cell biology, and disseminate within tissues (5, 9). In this sense, environmental persistence and host adaptation represent two facets of the same evolutionary strategy: survival under stress.

The remarkable ecological versatility of the *Mycobacterium* genus underpins its evolutionary transition from environmental saprophytes to opportunistic and obligate pathogens. Many traits that enable persistence in harsh environmental niches—such as slow growth, a hydrophobic and lipid-rich cell envelope, and robust biofilm formation—also confer intrinsic resistance to host immune defenses and antimicrobials (19, 22). Interactions with free-living amoebae (FLA) have likely served as an evolutionary crucible for intracellular adaptation, as NTM can survive within amoebal hosts, gaining protection from environmental stressors while preadapting to life inside macrophages (19, 23). Metabolic flexibility further supports this dual survival strategy: several environmental and opportunistic mycobacteria (including *M. smegmatis, M. neoaurum*, and *M. fortuitum*) are capable of degrading sterols and other complex organic compounds, and pathogenic species such as Mtb exploit this same metabolic machinery to utilize host cholesterol as a carbon source during infection (22, 24–28). Together, these ecological, physiological, and metabolic traits illustrate how the genus has been repeatedly shaped by environmental pressures, equipping mycobacteria with the adaptive versatility needed to thrive across the continuum from free-living saprophytes to highly specialized intracellular pathogens.

### 2.2 Coevolutionary pressures between mycobacteria and hosts

The transition from a generalist environmental lifestyle to a highly specific, host-adapted existence imposes intense selective pressures that drive diverse evolutionary trajectories within the *Mycobacterium* genus (29). Host environments exert intense selective forces that drive specific metabolic and genetic adaptations for long-term persistence. For instance, in a study by Vázquez et al. (30), experimental selection of *M. bovis* BCG in long-term macrophage culture resulted in adapted strains that exhibited improved glucose metabolism (i.e., a switch to glycolytic substrates), increased neutral lipid accumulation, and a lack of peptidoglycan production. Such adaptations correlated directly with increased survival within macrophages and prolonged residence in mice (30).

Obligate intracellular pathogens, such as members of the MTBC, inhabit stable, often sterile host environments, limiting opportunities for horizontal gene exchange and leading to a highly clonal population structure (29). These species evolve predominantly through gene loss and decay, a phenomenon known as reductive evolution, leading to genome downsizing (18, 29). *M. leprae* and *M. ulcerans* exemplify extreme cases of this trajectory. *M. leprae* possesses the highest proportion of pseudogenes known in any prokaryote or eukaryote, approximately 41% (18, 19, 31), resulting in dependence on the host for key nutrients due to the elimination of redundant biosynthetic pathways (31). Similarly, *M. ulcerans* evolved recently from the environmental species *M. marinum* through this process, becoming a niche-adapted specialist, evidenced by the proliferation of insertion sequences (IS*2404*, IS*2606*) and widespread chromosomal rearrangements (15).

Within-host evolution continues to shape pathogenic populations over shorter timescales, particularly under antimicrobial treatment (32). In the case of Mtb, the evolution and acquisition of drug resistance rely almost exclusively on chromosomal mutation and clonal spread, since this ancient pathogen lacks active horizontal gene transfer (HGT) and does not harbor mobile resistance elements (33–35). The Mtb genome shows no evidence of recombination, meaning that all resistance to antituberculosis drugs arises through spontaneously occurring mutations, typically single nucleotide polymorphisms (SNPs) (33–35). These variants emerge during within-host evolution, where the large bacterial population size [often exceeding 10^9^ colony-forming units (CFUs)] provides a substantial mutational reservoir (34, 35). Under antibiotic pressure, mutants that gain a fitness advantage expand and eventually become the dominant clone within the host before being transmitted onward (34, 35).

Drug resistance evolution in Mtb often follows a pattern of branched evolution, where multiple subpopulations carrying distinct mutations coexist during treatment (32). The spatiotemporal heterogeneity of antimicrobials and bacterial density within lesions, alongside phenotypic drug tolerance, influences this evolutionary path (32). Over time, purifying selection exerted by effective antimicrobials often leads to the dominance of a single, highly fit resistant clone—the “dominant lineage” model (32). Furthermore, recent genomic studies show that Mtb clinical isolates exhibit mutational signatures correlating with host immune environments, highlighting the host's direct role as an evolutionary filter (32).

### 2.3 Comparative genomics and evolutionary signatures of virulence

Genomic comparison studies are essential for elucidating mechanisms of pathogenicity and for revealing the roles of mobile elements, HGT, and accessory gene content in shaping virulence traits. The introduction of genetic material via HGT is a critical mechanism driving the speciation and virulence of some mycobacteria (4, 30, 36). In contrast, the MTBC shows no evidence of ongoing recombination or active HGT, so its pathogenic diversification has occurred primarily through vertical inheritance, gene loss, and spontaneous chromosomal mutations (33–35). In this sense, mobile genetic elements such as insertion sequences (IS) are key drivers of bacterial genome plasticity, virulence, persistence, and drug resistance (18, 37). For instance, *M. ulcerans* acquired a large plasmid encoding polyketide synthases responsible for producing the necrotic macrolide toxin, mycolactone (15, 30). Moreover, the acquisition of epigenetic modifiers (e.g., a putative DNA methylase, DpnM) in *M. abscessus* increased pathogenic potential and antibiotic resistance in specific clones (38).

The distribution of specific gene families across the genus reflects adaptation to distinct ecological niches. For instance, virulent mycobacteria show marked expansions of the PE/PPE protein families, duplication, and specialization of ESX (type VII secretion system), and enrichment in complex lipid biosynthetic pathways. These genomic patterns directly link virulence evolution to the pressures of intracellular survival and immune evasion (3, 9).

Parametric genomic analyses have further identified numerous regions of probable foreign origin, often termed genomic or pathogenicity islands (39). These foreign DNA fragments, accounting for roughly 4.5% (199 kb) of the Mtb genome, frequently contain genes with putative or documented virulence functions (39). Examples include the *Rv0986–Rv0988* operon, likely acquired from a γ-proteobacterium, which contributed to the emergence of the ancestral Mtb lineage as a successful intracellular pathogen (6). Other acquired regions code for critical adaptation factors, such as components required for anaerobic growth, reflecting adaptation for survival under reduced oxygen conditions, as found in granulomas (6). The *espACD* locus, which regulates secretion of the ESAT-6 virulence factor, also appears to have been laterally transferred into related species such as *Mycobacterium decipiens* (5).

In parallel, reductive evolution has been a defining feature of pathogenic specialization. The MTBC genome (4.4 Mb) is markedly smaller than that of its environmental relatives, such as *M. marinum* (6.6 Mb) and *M. kansasii* (6.4 Mb), reflecting the streamlining associated with an obligate intracellular lifestyle. This downsizing is thought to be an adaptation mechanism to the specialized niche of the mammalian host (6). Characteristic deletions, including the TbD1 region, which is lost in modern epidemic lineages (L2, L3, L4), have been linked to increased resistance to oxidative stress and hypoxia (9). Similarly, the loss of the *crtEIB* pigmentation locus in MTBC members signals adaptation away from environmental habitats and toward mammalian hosts (5). These deletions illustrate how genome contraction can serve as an adaptive response to a more specialized niche.

Finally, comparative analyses of virulence determinants underscore the refinement of secretion and surface-exposed systems during pathogenic evolution. The ESX pathways and the PE/PPE protein families have undergone repeated duplication and diversification (6). The ESX-1 system, a major virulence determinant required for macrophage spread and granuloma formation, is present in Mtb and in pathogenic slow-growing mycobacteria such as *M. marinum*, suggesting its acquisition was driven by selection for an intracellular niche (6, 17). Lineage-specific mutations within these systems continue to shape virulence phenotypes; for instance, variation in *esxW* has been associated with enhanced transmissibility in modern lineages, while truncations in *esxM* have been associated with altered macrophage migration and dissemination (9). Other mutations, such as deletions in *fadB4* that increase hydrophobicity and promote intracellular replication, further exemplify ongoing adaptive refinement (9).

Viewed through an evolutionary lens, the genus *Mycobacterium* encapsulates the continuum from environmental opportunism to obligate pathogenicity. This progressive adaptation to intracellular life provides not only a framework for understanding bacterial evolution but also a window into how ancient interactions with phagocytic hosts forged conserved innate immune mechanisms. The study of mycobacteria across diverse host systems thus reveals how microbial pressures have shaped the architecture and complexity of innate immunity.

## 3 Amoebae as ancient selective pressures

### 3.1 Ecological origins: amoebae as proto-macrophages and evolutionary training grounds

FLA are ubiquitous, cell-wall-free, unicellular eukaryotes found in environments like soil and water, and they feed on bacteria (40–42). Laboratory investigations commonly use the social soil amoeba *Dictyostelium discoideum* (Dd) and waterborne amoebae such as *Acanthamoeba castellanii, Acanthamoeba polyphaga*, and *Hartmannella vermiformis* (41, 43, 44). These amoebal groups demonstrate marked differences in their life cycles and responses to environmental stress (42, 45, 46). Social amoebae like Dd spend the majority of their existence as unicellular trophozoites, but in response to starvation or nutrient depletion, they initiate a multicellular developmental program (45). This process results in the formation of aggregates, a migrating slug, and ultimately a fruiting body containing individual spores in the sorus (45, 46). In contrast, solitary amoebae do not undergo multicellular development (40, 42). Instead, when exposed to adverse conditions like starvation, desiccation, or temperature shifts, they form highly resistant single-celled cysts. The cyst wall provides robust protection against environmental stress and can shelter internalized bacteria (40, 42, 46). A key distinction among FLA is their temperature tolerance. *Acanthamoeba* species can thrive at 32–37°C, a range that coincides with that of mammalian hosts (46), whereas most other FLA (including Dd) grow optimally between 15–25 °C and do not tolerate sustained higher temperatures. This thermotolerance makes *Acanthamoeba* one of the few environmental protozoan hosts capable of exerting selective pressure at mammalian-like temperatures, suggesting a natural training grounds for intracellular bacterial pathogens, including mycobacteria, allowing them to adapt to conserved eukaryotic processes found in macrophage-like environments (40, 46). These ecological constraints highlight the unique position of amoebae as environmental phagocytes, setting the stage for comparison with later-evolving hosts in which similar pressures shaped conserved defense strategies.

**Figure 2:**
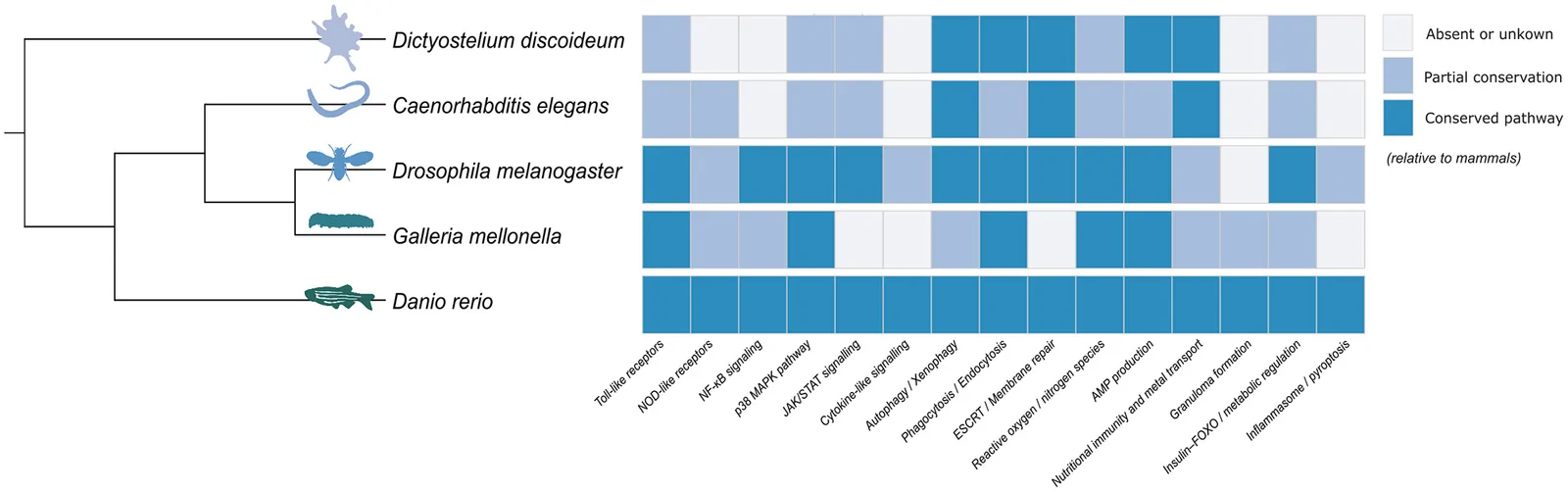
Conservation of innate immune pathways across model organisms used to study mycobacterial infection. The cladogram illustrates evolutionary relationships among the model organisms discussed in this review, and it was built using the Interactive Tree of Life (iTOL). The heatmap summarizes the presence and functional conservation of key innate immune pathways across the five experimental models discussed in this review. Pathway conservation is shown relative to mammals: white squares indicate pathway components that are absent or currently unknown, light blue squares indicate lack of the canonical pathway but presence of a functional and evolutionarily related system, and dark blue squares indicate components with structural and/or functional homology to the canonical pathway.

Within this framework, Dd has emerged as a powerful model for studying host–pathogen interactions. Often regarded as a “proto-macrophage,” Dd combines the simplicity of a unicellular organism with a conserved endocytic and signaling machinery that closely parallels that of animal phagocytes (10, 13, 47–51). As depicted in Figure 2, its ecological role as a professional phagocyte has driven the evolution of fundamental defense mechanisms conserved with those of animal innate immune cells, including macrophages and neutrophils (10, 48, 50, 52, 53). This conservation supports the hypothesis that amoebae served as an evolutionary “training ground” for intramacrophage pathogens such as mycobacteria (12, 49–51, 54).

Interactions between Dd and *M. marinum*, a close genetic relative of Mtb, illuminate several conserved cell-autonomous defense mechanisms (45, 50, 55–57). Amoebae engulf particles into phagocytic vacuoles, or phagosomes, which are normally intended to progress through an endosomal-lysosomal degradation pathway involving fusion with lysosomes and acquisition of enzymes, ultimately resulting in the intracellular digestion and destruction of the particle (45, 46). However, pathogenic mycobacteria have evolved mechanisms to subvert this endosomal-lysosomal maturation process to establish a mycobacteria-containing vacuole (MCV), within which they can proliferate (45, 46). Depending on the species and environmental context, interactions between mycobacteria and amoebae may result in bacterial killing, host cell death, or the establishment of a stable intracellular relationship, as seen with amoeba-resistant organisms (AROs) (42, 45, 46). In solitary amoebae such as *Acanthamoeba*, this intracellular persistence can be further reinforced during encystment, where surviving bacteria become enclosed within the cyst wall. This highly protective structure enhances resistance to desiccation and antimicrobial agents and acts as a “Trojan horse” that facilitates long-term environmental survival (58, 59).

### 3.2 Cellular immunity: conserved defense and evasion mechanisms

At the cellular level, Dd employs an extensive repertoire of ancient, cell-autonomous defense mechanisms that mirror those of mammalian phagocytes. A central component is the divalent cation transporter Nramp1 (Slc11a1), a homolog of the mammalian macrophage resistance protein NRAMP1 (48, 60, 61). When delivered to the phagosomal membrane, Nramp1 exports metal ions (mostly Fe^2+^ and Mn^2+^) and limits bacterial access to essential nutrients (62, 63). Thus, amoebae lacking this transporter are markedly more permissive to *M. avium* infection (60, 61, 64).

In addition to iron deprivation, Dd utilizes zinc intoxication as a form of nutritional immunity. By elevating intravacuolar Zn^2+^ concentrations (via ZIP-family importers and ZnT-family transporters that regulate intracellular zinc), the amoeba creates a toxic environment that restricts mycobacterial survival. Consistent with this, Dd mutants unable to increase intracellular zinc levels—such as those lacking key ZnT transporters—show significantly impaired *M. marinum* intracellular growth compared to wild-type hosts (65). To counteract host-imposed zinc stress, *M. marinum* and related species upregulate the efflux transporter CtpC, and deletion of *ctpC* severely impairs intracellular growth (65, 66). Together, these strategies illustrate that manipulating metal availability—by both deprivation and poisoning—constitutes an evolutionarily ancient antimicrobial defense. Similar principles of metal-based nutritional immunity reappear in more complex hosts, although diversified through additional regulatory layers.

Although it does not possess canonical antimicrobial peptides (AMPs), Dd expresses small secreted proteins with antimicrobial activity, functionally analogous [i.e., saposin-like proteins (SAPLIPs), also called amoebapore-like peptides (Apls)] (67, 68). In addition, Dd exhibits a combination of conserved and lineage-specific elements in nutritional immunity and metal transport: while core pathways for iron and zinc homeostasis are evolutionarily conserved with mammals, other components represent amoeba-specific adaptations (60, 61, 69).

Phagocytic uptake in Dd also depends on conserved membrane receptors, such as the lysosomal membrane proteins LmpA and LmpB, which are homologous to the mammalian LIMP-2 and CD36 scavenger receptors (48). These proteins mediate bacterial internalization and regulate phagosomal acidification and proteolysis; their disruption increases susceptibility to *M. marinum* infection (48). Once internalized, pathogenic mycobacteria deploy the ESX-1 secretion system to damage the MCV through membranolytic effectors such as EsxA and phthiocerol dimycocerosate (PDIM), enabling partial escape into the cytosol (45, 54, 66, 70, 71). The host responds by coordinating the activation of membrane repair pathways involving the endosomal sorting complex required for transport (ESCRT) and the autophagy machinery (56, 70–72). The ubiquitin ligase TrafE orchestrates recruitment of these systems to damaged membranes, with ESCRT repairing small perforations while autophagy patches larger lesions (56, 70). Disruption of either process accelerates cytosolic escape of *M. marinum*, underscoring their cooperative importance in containing infection (56).

Intriguingly, mycobacteria exploit these same defenses to their advantage. *M. marinum* induces autophagy gene expression and recruits autophagosomes to the MCV but simultaneously inhibits autophagic flux in an ESX-1– and TORC1-dependent manner, thereby preventing bacterial degradation (72, 73). Cytoskeletal remodeling further shapes the infection outcome. Host proteins such as the small GTPase RacH and flotillin-like vacuolins regulate actin polymerization and vacuolar dynamics; loss of vacuolin isoforms restricts bacterial growth, whereas RacH deficiency enhances susceptibility (74, 75). Moreover, the ESX-1 system, along with EsxA, is also required for the bacteria's eventual exit from the amoeba, which occurs via a nonlytic, actin-dependent mechanism known as ejectosome-mediated ejection (47, 75, 76). Notably, the autophagic machinery actively ensures this nonlytic transmission process (13, 73). This non-destructive egress parallels actin-based cell-to-cell spread in macrophages, highlighting the evolutionary conservation of intracellular infection strategies. Thus, amoebae already display the core logic of intracellular confrontation that is reiterated, refined, and contextually expanded across invertebrate and vertebrate hosts.

### 3.3 Ancestral signaling and evolutionary insights

Despite lacking canonical Toll-like receptors (TLRs), Dd expresses a large family of transmembrane proteins containing leucine-rich repeats (LRRs) that function analogously as pattern-recognition molecules (69, 77). Other components of its innate machinery, including nucleotide-binding domain–like receptors (NLRs) and autophagy-related proteins, demonstrate evolutionary conservation with animal innate immune signaling pathways (78–82). The highly conserved nature of these host cell defense pathways, which mirror those in mammalian macrophages, validates the concept of Dd as a model for cell-autonomous defenses (10, 13, 45, 48, 50). Moreover, although ancestral, the reactive oxygen species (ROS) machinery of Dd is functionally equivalent to the vertebrate oxidative burst (83, 84). The presence of these conserved effectors in amoebae provides a baseline for interpreting how later organisms diversified but retained the fundamental architecture of these responses.

The similarities extend beyond general cellular processes to include specific counter-strategies, such as the ESX-1-mediated phagosome damage and the host's use of ESCRT/autophagy repair mechanisms -including core components such as ESCRT-III subunits (e.g., Vps32/CHMP4), ESCRT-I (e.g., Tsg101), TrafE, and accessory proteins like ALIX- and nutritional immunity via metal trafficking (56, 60, 65, 71, 73). The shared virulence strategies and host responses suggest that the interaction between mycobacteria and professional phagocytes originated during their ancient co-evolution in environmental niches (13, 49, 51, 71). Furthermore, Dd utilizes a collaborative exclusion mechanism during its multicellular developmental cycle to exclude infected cells, representing an ancient, multicellular form of innate immunity and clearance (10).

However, Dd also reveals evolutionary divergences that highlight the evolution of immunity. Unlike metazoans, Dd lacks a homolog of the p38 mitogen-activated protein kinase (MAPK) pathway (85). However, it possesses p38-like stress-activated protein kinase (SAPKα) and ERK1/2 cascades that regulate stress responses, cytoskeletal dynamics, and chemotaxis via G protein-coupled rather than receptor tyrosine kinase signaling (86–88). Similarly, while the insulin–FOXO signaling axis is functionally conserved, its architecture in Dd is only partially homologous: the organism retains the ancestral PI3K–Akt–TOR nutrient-sensing module. Still, it lacks the dedicated insulin/IGF ligands and receptors that define the metazoan pathway (89). Moreover, Dd has conserved biochemical systems for ROS, homologous to those in mammals, while lacking a canonical Nitric Oxide Synthase (NOS) enzyme homologous to those found in mammals (90–92).

Finally, at the effector level, Dd can produce DNA extracellular traps (ETs), a mechanism previously thought to be restricted to multicellular organisms, demonstrating that this antimicrobial strategy evolved well before the origin of metazoans (14). DNA ET formation in Dd occurs only during the multicellular slug stage, where specialized Sentinel cells function as an ancient innate immune system capable of releasing DNA ETs. In contrast, Dd lacks canonical inflammasome components and pyroptotic cell-death pathways (93). These absences also clarify which immune innovations emerged later in metazoans, allowing direct comparison with the expanded innate repertoires found in *C. elegans, D. melanogaster*, and zebrafish.

Together, these features position Dd as a living window into the evolutionary origins of innate immunity. Its genome encodes conserved effectors for phagocytosis, metal trafficking, autophagy, and ROS-mediated killing, yet lacks the complex cytokine networks and multicellular coordination that characterize animal immunity. This balance of conservation and simplicity makes Dd an invaluable comparative model for understanding how ancient, cell-autonomous defenses were progressively co-opted and expanded into the integrated innate immune systems of metazoans.

## 4 Non-vertebrate metazoan models

### 4.1 Caenorhabditis elegans

*C. elegans* is a ubiquitous nematode that lives in soil and feeds on bacteria and is a widely used model organism for studying genetics, immunology, and host-pathogen interactions (94–97). Several bacterial species—including *Escherichia coli, Pseudomonas aeruginosa, Staphylococcus aureus*, and *Enterococcus faecalis*—cause intestinal infection in *C. elegans*, leading to pathogen proliferation in the gut lumen, epithelial damage, and nematode death (94, 98). As an invertebrate, *C. elegans* relies exclusively on its innate immune system and lacks both adaptive immunity and professional immune cells such as macrophages (95, 96, 99). Its defense, therefore, depends mainly on epithelial immunity, particularly in the intestine, which is composed of only 20 non-renewable cells that act as both physical and immunological barriers (11, 96). Compared with unicellular amoebae, *C. elegans* thus illustrates how ancient cell-autonomous defenses are redeployed within a simple metazoan tissue context.

The relationship between *C. elegans* and mycobacteria provides a crucial model for studying the pathogenesis of TB and other mycobacterial infections (97). For instance, infection with pathogenic *M. marinum* results in high morbidity and mortality (e.g., >80% mortality within 24 h), whereas the non-pathogenic *M. smegmatis* causes minimal mortality (< 15%) (97). Pathogenic *M. marinum* causes irreversible pathological changes in the nematode, including loss of pigmentation and “bagging” (embryo retention leading to the death of the adult worm, which is a stress response) (97). These conserved pathological outcomes and host responses indicate that the underlying pathogenic mechanisms exploited by *M. marinum* in the simple host are relevant to mammalian infection (Figure 2) (97). Similar virulence hierarchies and pathological signatures for *M. marinum* vs. non-pathogenic species are also observed in amoebae, flies, and zebrafish, underscoring that core mycobacterial strategies are conserved across very distant hosts.

#### 4.1.1 Epithelial immunity: the p38 MAPK pathway and conserved stress responses

The core defense mechanism against mycobacteria in *C. elegans* is governed by the conserved p38 mitogen-activated protein kinase (MAPK) pathway—rather than direct microbial recognition through TLRs—centered around the protein PMK-1 (97). *C. elegans* detects mycobacteria by activating TLR-independent innate immune pathways, primarily via the PMK-1/p38 MAPK cascade, and by leveraging unique receptors and neuronal circuits that enable recognition of microbial patterns and metabolites (96, 100). PMK-1 is normally localized in the cytoplasm of intestinal cells and neurons, but upon stress or infection, it becomes phosphorylated and translocates to the nucleus (101, 102). PMK-1 does not directly sense pathogens; instead, it functions as the central kinase downstream of the adaptor TIR-1/SARM, which acts as an upstream sensor and NADase required for pathway activation (103–105). This module is orthologous to the ASK1/MKK3/6/p38 MAPK pathway found in mammals, representing an evolutionarily conserved module used in defense against pathogenic attack (96, 99, 106). PMK-1 regulates the basal and inducible expression of antimicrobial effectors, including secreted C-type lectins and CUB-like proteins (99). Loss-of-function mutants in *pmk-1* are hypersusceptible to *M. marinum*, exhibiting complete mortality during infection, demonstrating the indispensability of this pathway for nematode immunity (97).

Although the PMK-1 module functions analogously to the mammalian p38 MAPK cascade, the *C. elegans* genome lacks several other canonical components of the innate immune system. Notably, it does not encode any NF-κB homologs, nor the IKK complex (IKKα, IKKβ, NEMO/IKKγ) or IκB inhibitors that regulate NF-κB activation. Consequently, *C. elegans* lacks the canonical Toll → MyD88 → IRAK → TRAF → IKK → NF-κB signaling cascade that, in most metazoans, regulates not only AMP induction but also broader stress-response programs controlling inflammation, cell survival, proliferation, and both innate and adaptive immune functions (96). Similarly, although *C. elegans* possesses two STAT homologs (STA-1 and STA-2), it lacks both the JAK kinases and the canonical upstream cytokine/receptor signaling found in mammals (107, 108).

Pathogenic mycobacteria appear to exploit this ancient defense pathway to enhance their virulence. *M. marinum* infection suppresses PMK-1 activation through the host MAPK phosphatase VHP-1, a negative regulator of the p38 MAPK pathway (97). Genetic loss of *vhp-1* restores PMK-1 activity and significantly increases host survival following infection, whereas wild-type animals succumb rapidly (97). This inverse relationship between MAPK activation and susceptibility suggests that *M. marinum* manipulates host phosphatase activity to subvert immunity- an effect consistent with MAPK modulation observed in mammalian macrophages (97).

Unlike mammals, which have multiple inflammasome complexes that activate inflammatory caspases, such as caspase-1, to induce pyroptosis via gasdermin proteins, *C. elegans* has a simpler caspase system, primarily involving CED-3 for apoptosis (109–111). Despite this simplicity, the nematode mounts robust innate responses through conserved effector mechanisms. *C. elegans* produces AMPs that are analogous to, but not strictly canonical with, those found in mammals. It has several families of AMPs, including caenopores (saposin-like proteins), defensin-like antibacterial factors (ABFs), and other peptides such as neuropeptide-like and caenacin peptides (112, 113). These antimicrobial peptides, along with PMK-1–regulated lectins and CUB-like proteins, collectively form a significant humoral barrier to infection in the absence of inflammatory cytokines.

Pathogenic *M. marinum* appears to exploit this ancient defense pathway to enhance its virulence. Infection suppresses PMK-1 activation through the host MAPK phosphatase VHP-1, a negative regulator of the p38 MAPK pathway (97). Genetic loss of *vhp*-*1* restores PMK-1 activity and significantly increases host survival following infection, whereas wild-type animals succumb rapidly (97). This inverse relationship between MAPK activation and susceptibility suggests that *M. marinum* manipulates host phosphatase activity to subvert immunity—an effect consistent with MAPK modulation observed in mammalian macrophages (97).

Downstream of PMK-1, the transcription factor SKN-1 -the nematode ortholog of mammalian Nrf1/Nrf2- mediates oxidative stress and detoxification responses (97). The DUOX–SKN-1 is functionally conserved but non-phagocytic (114, 115), and studies show that SKN-1 activation protects the host from self-inflicted oxidative damage generated during the antimicrobial response. *M. marinum*-infected *skn-1* mutants exhibit exacerbated pathology and accelerated mortality, confirming SKN-1's role as a critical downstream effector of PMK-1-mediated protection (96, 97, 116).

Because *C. elegans* lacks phagocytic immune cells, antimicrobial defense depends on epithelial sensing rather than intracellular phagosome-mediated pathways, with the intestinal epithelium detecting and responding to microbes through conserved pattern-recognition-like mechanisms. Instead of mounting inflammatory reactions, the nematode responds through transcriptional reprogramming that balances immune activation with metabolic cost, maintaining homeostasis in the context of a dynamic microbiota. Nevertheless, pathogenic *M. marinum* can persist within the gut and disrupt intestinal villi, potentially through mechanisms that manipulate actin dynamics in a manner reminiscent of mycobacterial infection in macrophages (97).

Autophagy pathways in *C. elegans* are highly conserved with those in mammals. The nematode encodes orthologs of canonical autophagy genes such as *Atg1–10, Atg12, Atg16*, and *Atg18*, as well as homologs of *Atg4, Atg8*, and *Atg16*. These components participate in the same fundamental processes of autophagosome formation, substrate degradation, and regulation as their mammalian counterparts. Additionally, key transcriptional regulatory networks, such as the Krüppel-like transcription factors, modulate autophagy in both *C. elegans* and mammals (117–119).

*C. elegans* also possesses conserved ESCRT-mediated membrane repair mechanisms similar to mammals. Following plasma membrane damage, ESCRT-III components such as VPS-32.1 (a CHMP4B ortholog) and VPS-4 (VPS4A/B ortholog) are recruited to wound sites to mediate membrane repair. These complexes facilitate membrane remodeling by recruiting additional proteins, such as TSP-15, Syntaxin-2, and EFF-1, which are essential for wound closure and repair (120–122).

These pathways also interface with mitochondrial and oxidative stress sensors, integrating metabolic and immune regulation in a system that predates cytokine-based communication. While other conserved pathways (e.g., the DAF-2/DAF-16 insulin-like signaling cascade and the *tol-1* TLR homolog) also contribute to nematode immunity (96, 99), they do not appear to play a significant role in mycobacterial susceptibility (97). Finally, metal homeostasis also represents an evolutionarily conserved arm of innate defense. *C. elegans* regulates intracellular levels of divalent cations such as Fe^2+^, Zn^2+^, and Mn^2+^ through conserved transporters, limiting microbial access to essential micronutrients (123).

Thus, the *C. elegans–Mycobacterium* interaction model bridges unicellular and multicellular host systems, offering insight into how early metazoans combined cell-autonomous defenses with coordinated tissue responses. This architecture likely represents a key evolutionary transition toward the more complex, multicellular innate immune networks observed in arthropods and vertebrates.

### 4.2 Drosophila melanogaster

The fruit fly *D. melanogaster* has emerged as a crucial invertebrate model for dissecting the fundamental mechanisms of innate immunity and host-pathogen interactions, particularly in the context of mycobacterial infections and TB pathogenesis (124–128). The fly's reliance exclusively on its innate immune system, coupled with its genetic tractability and the high degree of conservation of immunological and disease-related genes with mammals (approximately 75% of human disease-causing genes have homologs in the fly genome), makes it highly valuable for this comparative approach (124–131). Infection studies primarily utilize *M. marinum*, a close genetic relative of Mtb that causes a TB-like disease in cold-blooded animals, as a model for TB (125, 128, 129, 132). More recently, emerging pathogens such as *M. abscessus* have also been used to study mechanisms of virulence and host adaptation (126, 128, 133). In this sense, *D. melanogaster* occupies an intermediate position between nematodes and vertebrates, retaining an innate-only immune system but adding professional phagocytes and complex organ physiology that more closely approximate mammalian infection biology.

#### 4.2.1 Cellular immunity: phagocytes, entry, and conserved evasion mechanisms

The fly's cellular immune system is composed of specialized phagocytes known as hemocytes, the majority of which are plasmatocytes that function analogously to mammalian macrophages (127, 128). During *M. marinum* infection, bacteria initially proliferate within these phagocytes before disseminating through the hemolymph, producing a lethal systemic infection that mirrors key aspects of vertebrate TB (125, 129, 132). Genome-wide RNA interference (RNAi) screens in *Drosophila* S2 macrophage-like cells—which recapitulate hemocyte behavior *in vivo*—have been pivotal in identifying host determinants of mycobacterial infection. These studies revealed that S2 cells effectively restrict non-pathogenic species, such as *M. smegmatis*, while permitting intracellular growth of pathogenic mycobacteria, including *M. fortuitum* and *M. marinum* (127, 128, 134, 135).

Among the host factors identified, Peste (Pes), a CD36 family member/Class B Scavenger Receptor (SR), plays a key role in the uptake of *M. fortuitum, M. smegmatis*, and *Listeria monocytogenes*, but not *E. coli* or *S. aureus* (128, 134, 135). The fact that mammalian class B SRs (SR-BI and SR-BII) uniquely mediate *M. fortuitum* uptake into non-phagocytic cells suggests a conserved role for Class B SRs in pattern recognition and innate immunity against mycobacteria (135).

Once inside the host cell, pathogenic mycobacteria employ conserved strategies to evade intracellular defenses. In *Drosophila* hemocytes, *M. marinum* blocks vacuole acidification, preventing phagosome maturation and enabling intracellular survival (132). This subversion mirrors the behavior of Mtb in mammalian macrophages, suggesting that mycobacteria exploit evolutionarily conserved cellular processes for persistence (135).

An essential aspect of this defense interplay involves DUOX-derived ROS, which act as antimicrobial effectors. In *Drosophila*, the interplay between NADPH oxidase–generated ROS and autophagy in macrophage-like hemocytes is evolutionarily conserved with that of mammalian macrophages (100–102). These ROS serve dual roles—direct bacterial killing and modulation of autophagic flux—demonstrating the ancient coupling between oxidative stress and intracellular pathogen control.

Similar parallels are observed with *M. abscessus*, which exhibits a highly virulent phenotype in *Drosophila* infection models. The bacterium resists innate cytotoxic mechanisms by surviving lysis and caspase-dependent apoptotic cell death of infected hemocytes (127, 133). These cytotoxic events are mediated by specialized hemocyte subsets named thanacytes, whose apoptosis typically contributes to bacterial clearance. However, *M. abscessus* withstands this defense, escaping from lysed cells to disseminate systemically and cause bacteremia and death in the fly (127, 133). This ability to resist hemocyte cytotoxicity mirrors that of Mtb in vertebrate hosts and may explain the exceptional pathogenicity of *M. abscessus* among rapidly growing mycobacteria (127, 133).

In parallel, *D. melanogaster* possesses conserved ESCRT-mediated membrane repair and remodeling mechanisms similar to those in mammals. ESCRT-III components such as Shrub (CHMP4B ortholog) regulate the recycling and degradation of junctional proteins to maintain epithelial integrity and compensate for barrier defects (136, 137). This system likely contributes to cellular recovery following mycobacterial-induced damage, supporting host survival during infection.

#### 4.2.2 Humoral immunity: the paradox of pattern recognition

The humoral immune response in *Drosophila* relies on the Toll and IMD pathways, which activate NF-kB-related transcription factors (Dorsal/Dif and Relish, respectively) to induce the production of AMPs (126, 127, 131). Beyond these canonical surface recognition systems, the fly also possesses a cytosolic surveillance layer that parallels aspects of mammalian inflammasome biology (Figure 2). Although it lacks a true inflammasome, *Drosophila* retains several ancestral components of innate immune sensing (138). Its genome encodes NLR-like proteins such as NAIP and the Apaf-1 ortholog CED-4, which function as cytosolic danger sensors, but lacks gasdermin-family pore formers required for pyroptosis (139, 140). Instead, the caspase-8–like protease Dredd acts downstream of the IMD pathway to activate Relish, fulfilling an analogous role in executing immune and apoptotic responses (141).

Intriguingly, early studies suggested that *M. marinum* and *M. smegmatis* induced minimal AMP responses, though the mechanisms underlying this weak activation remain unclear (128, 132). Furthermore, flies carrying mutations in either the Toll or IMD pathways showed wild-type sensitivity to lethal *M. marinum* infection (128). This suggested that either the fly failed to recognize mycobacteria as invaders, or that a mycobacterial component actively blocked NF-kB activation (132).

In contrast to *M. marinum*, infection with the fast-growing *M. abscessus* elicits strong Toll pathway activation (126–128). *M. abscessus* induces increased levels of AMP transcripts associated with both the Toll pathway (e.g., Metchnikowin) and the IMD pathway (e.g., Attacin-A and Diptericin) (126, 127). This AMP induction is often observed later in the infection (e.g., peak at 3–4 days post-infection) (126, 127, 142). Loss-of-function Toll pathway mutants showed increased mortality following *M. abscessus* infection, indicating that this pathway contributes to resistance (126, 142).

The reliance on the Toll pathway is paradoxical given that mycobacterial peptidoglycan contains meso-diaminopimelic acid (m-DAP), which typically signals via the IMD pathway. This delayed and unusual recognition suggests that the mycobacterial membrane shields the peptidoglycan from efficient detection (128). It is hypothesized that an alternative microbial-associated molecular pattern, such as a glycan or other factor in the bacterial membrane, is recognized by receptors that activate the Toll pathway, or that detection is triggered indirectly by the severe tissue damage caused by the infection (127, 128).

#### 4.2.3 Stress and signaling pathways in host defense

The p38 MAPK pathway in *Drosophila* is both structurally and functionally conserved with that in mammals. In *Drosophila*, there are three p38 MAPK orthologs (i.e., p38a, p38b, and p38c), regulated by upstream MAP kinase kinase kinases (MAPKKKs) such as D-MEKK1 and MAP kinase kinases (MAPKKs) such as D-MKK3/4 (encoded by *licorne*). These kinases regulate the phosphorylation and activation of p38 MAPKs, which, in turn, regulate a broad set of physiological responses, including stress tolerance and immune defense against pathogens (143, 144). This conservation mirrors the PMK-1–based p38 MAPK defense module in *C. elegans* and underscores a shared ancestral signaling architecture linking stress and immunity.

#### 4.2.4 Metabolic dysregulation: a conserved pathology

Studies in *Drosophila* have provided crucial insight into the metabolic consequences of mycobacterial infection, revealing that pathogen-induced metabolic dysregulation is an evolutionarily conserved feature of host–pathogen interactions. Beyond the classical dichotomy of resistance (i.e., pathogen clearance) vs. tolerance (i.e., damage limitation), the fly model has been instrumental in dissecting how systemic metabolism is rewired during infection and how this contributes to disease pathophysiology (142, 145). Mycobacteria such as *M. marinum* and *M. abscessus* perturb host metabolism by hijacking central metabolic pathways: they manipulate amino acid transport (e.g., *M. abscessus* requires the asparagine transporter MAB_1132c to induce metabolic imbalance), disrupt systemic insulin signaling to suppress anabolic processes, and reprogram lipid and carbohydrate metabolism in the fat body. Immune–metabolic crosstalk further amplifies this shift, as microbial signals activate Toll, IMD, and JNK pathways, reinforcing the switch from storage to catabolism (128, 142, 146, 147).

Infection with *M. marinum* induces a wasting syndrome in flies that closely parallels cachexia observed in human TB and sepsis. Infected flies progressively lose lipid and glycogen stores and develop hyperglycemia, reflecting a chronic energy imbalance driven by persistent immune activation (125, 128, 129, 132, 143). This infection-induced wasting is not simply a byproduct of pathogen burden but rather a maladaptive host response, suggesting that conserved metabolic pathways are co-opted during infection to prioritize immune function at the expense of energy homeostasis.

At the molecular level, *M. marinum* infection disrupts the insulin signaling cascade, a central regulator of systemic metabolism. The infection leads to reduced Akt kinase activity, which, in turn, results in constitutive activation of the transcription factor FOXO (125, 128, 129). Activated FOXO drives the transcription of catabolic and stress genes and suppresses anabolic metabolism, accelerating the depletion of fat and glycogen stores. Remarkably, *foxo* mutant flies display attenuated wasting and survive longer following infection than wild-type flies, underscoring the causal link between immune-driven FOXO activation and metabolic deterioration (125, 128). These findings demonstrate that the Akt–FOXO axis represents a deeply conserved interface between nutrient sensing and immune activation, shaping host tolerance and survival outcomes across species.

Further insight into this immune–metabolic crosstalk has come from the identification of myocyte enhancer factor 2 (MEF2) as a pivotal transcriptional switch in the fly fat body, a tissue functionally analogous to the mammalian liver and adipose system (145). Under normal conditions, phosphorylated MEF2 promotes anabolic metabolism by activating the transcription of lipogenic and glycogenic enzymes. Upon infection, however, MEF2 loses its phosphorylation-dependent conformation and binds an alternative DNA motif, driving the expression of AMPs instead of metabolic genes (145). This functional switch from anabolism to immune activation enables a robust antimicrobial response but simultaneously suppresses energy storage pathways, contributing to the metabolic collapse observed in persistent infections.

Collectively, these observations demonstrate that infection-induced metabolic reprogramming is not merely a symptom but an adaptive, evolutionarily conserved aspect of host defense. By reallocating energetic resources toward immunity, the host enhances short-term survival at the cost of long-term metabolic stability. The parallels between wasting and insulin pathway dysregulation in flies and in human TB highlight the fundamental conservation of immune–metabolic trade-offs that underpin disease pathology across metazoans.

#### 4.2.5 Intersections with autophagy and nutrient sensing

Mycobacterial infection in *Drosophila* has revealed that the interplay between innate immunity, autophagy, and metabolism is deeply conserved across metazoans. These pathways, originally studied for their roles in cellular homeostasis, have emerged as central to antimicrobial defense, reflecting the evolutionary coupling of stress responses and immunity.

One key example is the role of PARKIN, the ubiquitin ligase encoded by the *park* gene, which is homologous to the human *PARK2* associated with Parkinson's disease. Beyond its well-known function in mitophagy (i.e., the selective degradation of damaged mitochondria), PARKIN is now recognized as an essential component of innate immune defense. *Parkin*-deficient flies, like *Park2*-deficient mice, exhibit increased susceptibility to a variety of intracellular bacterial infections, including mycobacteria (130, 204). This shared phenotype underscores an ancient functional link between mitochondrial quality control and resistance to intracellular pathogens.

Mycobacterial challenge also reveals crosstalk between cytokine signaling, lipid metabolism, and autophagy. During *M. marinum* infection, *Drosophila* macrophage-like cells upregulate *upd3*, a cytokine analogous to mammalian IL-6, which activates the conserved JAK/STAT signaling cascade. This pathway, in turn, represses transcription of the autophagy-related gene Atg2, whose expression is protective against infection. ATG2 limits the accumulation of large lipid droplets that mycobacteria exploit as nutrient reservoirs, thereby restricting bacterial growth (128, 205). Thus, by suppressing ATG2, *M. marinum* effectively manipulates a conserved cytokine–autophagy axis to remodel host lipid metabolism for its benefit, a strategy also observed in mammalian macrophages.

Mitochondrial biogenesis and energy metabolism are likewise intertwined with antimicrobial immunity. The *Drosophila* homolog of PPARGC1A (PGC-1α), known as Spargel, orchestrates mitochondrial function downstream of AMP-activated protein kinase (AMPK). Flies lacking *spargel* are highly susceptible to *M. marinum* infection, demonstrating that the AMPK–PGC-1α axis, which controls both mitochondrial homeostasis and autophagic flux, is required for effective innate defense against mycobacteria (148).

Finally, studies with *M. abscessus* highlight how the pathogen's metabolic state influences host immune responses. Mutants with impaired asparagine transport display reduced virulence in *Drosophila*, delaying host mortality despite maintaining similar bacterial loads. This phenotype is accompanied by diminished AMP gene expression and reduced interference with systemic insulin signaling mediated by the interleukin-like cytokines Upd2 and Upd3. These findings suggest that the pathogen's metabolic status modulates host tolerance (damage limitation) rather than resistance (pathogen clearance), revealing an ancient metabolic dialogue that shapes infection outcomes across phyla (142).

Overall, the *D. melanogaster* model reveals that mycobacteria have evolved strategies to subvert both ancient cellular defense mechanisms (via conserved phagocytic evasion and resistance to cytotoxicity) and humoral immunity (via suppression/misdirection of AMP production) (128). Mycobacterial infection drives conserved pathological consequences, particularly metabolic wasting, by dysregulating ancient signaling pathways (Akt/FOXO, MEF2, JAK/STAT) conserved across metazoan evolution (82, 125, 128, 129, 145, 149). Together, these observations position *Drosophila* as a key model for uncovering how cellular metabolism, autophagy, and innate immunity have co-evolved as integrated networks.

### 4.3 Galleria mellonella

The larvae of the greater wax moth, *Galleria mellonella*, have emerged as a powerful non-mammalian host model for studying mycobacterial pathogenesis and innate immunity (150, 151). This system is cost-effective, ethically favorable, and physiologically relevant, relying exclusively on innate immunity and thus eliminating the confounding influence of adaptive responses (150–152).

*G. mellonella* can be infected with virulent members of the MTBC, including Mtb H37Rv and *M. bovis* BCG (153–155), as well as numerous NTM like *M. abscessus, M. marinum*, and *M. fortuitum* (154–158). Furthermore, *G. mellonella* experiments can be performed at 37 °C, the optimal temperature for many human pathogens, allowing for the study of mycobacterial physiology under conditions relevant to human disease (150, 154). This versatility, especially the ability to use virulent MTBC strains, provides an advantage over models like *Drosophila* and zebrafish, which often rely on surrogates such as *M. marinum* (151, 154). Together with *Drosophila* and zebrafish, *G. mellonella* therefore helps span the continuum from invertebrate to vertebrate hosts, enabling direct comparison of mycobacterial virulence strategies and host defenses across increasing levels of immune and anatomical complexity.

#### 4.3.1 Conserved innate immune mechanisms

The innate immune system of *G. mellonella* comprises both cellular and humoral components that parallel mammalian defenses in organization and function (Figure 2) (150, 151). The cellular arm is mediated by hemocytes, which function analogously to mammalian macrophages and neutrophils (150–152). Hemocytes are responsible for phagocytosis of invading pathogens, but this response is often insufficient to fully contain virulent mycobacteria (153). Studies using *M. abscessus* have demonstrated that the bacterium proliferates within infected phagocytic cells (159), and its capacity to survive the lysis and caspase-dependent apoptotic death of infected phagocytes is a virulence trait shared with strict pathogenic mycobacteria, such as Mtb (127).

A key evolutionary insight provided by *G. mellonella* is its response to mycobacterial sequestration, characterized by the formation of granuloma-like structures (GLS), also referred to as nodules or encapsulations (150–153, 160). GLS develop rapidly, sometimes within 24 h of challenge with *M. bovis* BCG, as hemocytes aggregate to sequester the bacteria (152). However, when infected with Mtb H37Rv, these structures eventually fail to restrain bacterial growth, leading to disseminated infection and host death (153). During this process, infected hemocytes accumulate lipid bodies rich in triacylglycerides (TAGs) (152). This mirrors the lipid accumulation seen in foamy macrophages within human granulomas, which serve as nutrient reservoirs that support mycobacterial persistence. This response represents an evolutionarily conserved attempt to contain infection, directly analogous to granuloma formation in vertebrate TB (152, 153). Thus, *G. mellonella* recapitulates key pathological and metabolic aspects of chronic TB, including the emergence of a lipid-rich environment conducive to bacterial dormancy and survival, although it also presents limitations.

At the molecular level, several immune pathways and effector mechanisms in *G. mellonella* exhibit strong evolutionary parallels with those in mammals. The TLR pathway is functionally analogous to the mammalian TLR system, mediating pathogen recognition and immune activation, although detailed molecular conservation is partial (155, 161). The p38 MAPK signaling cascade is also conserved and participates in immune regulation and stress responses during infection; infection with pathogens such as *Bacillus thuringiensis* induces phosphorylation and activation of p38 MAPK in the fat body and hemocytes (162).

ROS production represents another conserved antimicrobial defense. The core function of ROS-mediated microbial killing and associated enzymatic activity is strongly conserved between *G. mellonella* and mammals (163, 164), supporting a shared oxidative strategy for microbial control.

Autophagy processes in *G. mellonella* also show deep conservation with mammals, particularly in fundamental mechanisms involving Atg8 (mammalian LC3 ortholog), ubiquitylation, and autophagosome formation (165–167). Macroautophagy and its regulatory processes appear analogous in both systems, reinforcing autophagy as a core, ancient defense against intracellular pathogens.

Although no published studies explicitly describe ESCRT-mediated membrane repair in *G. mellonella*, this pathway is likely conserved, given its universal role in eukaryotic membrane integrity. Conversely, there is no direct evidence that *G. mellonella* possesses inflammasome complexes or pyroptosis mechanisms analogous to those found in mammals, suggesting that inflammatory cell death pathways may have diverged or are absent in this lineage.

#### 4.3.2 Humoral defense and antimicrobial effectors

The humoral immune response of *G. mellonella* complements its cellular defenses through the secretion of AMPs, complement-like proteins, ROS, and reactive nitrogen species (RNS) into the hemolymph (150, 152). Proteomic analyses of larvae infected with *M. bovis* BCG have identified the induction of several immune effectors, including cecropins and gloverins, which contribute to bacterial clearance (155). The induction of cecropins is particularly noteworthy from an evolutionary perspective. These peptides share the α-helical structural motif of the human cathelicidin LL-37 (hCAP-18), an AMP essential for innate resistance to Mtb (155). This structural and functional homology suggests that the use of α-helical peptides against mycobacteria is an ancient and conserved antimicrobial strategy across metazoans.

Additionally, *G. mellonella* infection activates the melanization cascade, functionally analogous to the mammalian complement system, and induces Hemolin, an opsonin-like protein that binds to mycobacteria and promotes phagocytosis. This further reflects conserved molecular logic in pathogen recognition (150, 155).

#### 4.3.3 Evolutionary relevance and virulence modeling

Beyond its mechanistic parallels, *G. mellonella* has proven instrumental in probing mycobacterial virulence and host adaptation. For instance, the deletion of the acyltransferase gene *mbtK* in *M. marinum*, required for the production of virulence lipids PDIM and phenolic glycolipid (PGL), results in attenuated infection in *G. mellonella*, confirming the model's suitability for virulence factor identification (66, 168). Phenotypic variation among NTM species is also faithfully recapitulated: rough colony variants (RCVs) of *M. abscessus* and *M. fortuitum* exhibit higher virulence and lower larval survival than smooth variants (SCVs), paralleling macrophage infection outcomes *in vitro* (158). Moreover, co-infection studies reveal that *M. abscessus* can suppress immune responses to *P. aeruginosa*, particularly by inhibiting melanization, thereby reducing host survival (169).

Finally, *G. mellonella* exhibits evidence of immune priming, a form of innate immune memory whereby prior exposure to sublethal infection or stress enhances survival upon subsequent challenge (150, 154). This response parallels trained immunity in mammals, particularly the long-lasting reprogramming of innate cells induced by *M. bovis* BCG vaccination (151). The ability to study immune memory and metabolic adaptation in a genetically tractable, innate-only organism positions *G. mellonella* as a pivotal model for understanding the evolutionary continuity of mycobacterial virulence and host tolerance mechanisms (150). However, a practical limitation of the system is that experimental infection is restricted to the larval stage, while in *Drosophila* infection can be studied in adult flies, allowing assessment of systemic physiology and survival in a fully developed organism (170, 171).

## 5 Zebrafish: a vertebrate model of innate immunity and granulomatous infection

The zebrafish (*Danio rerio*) is a readily available and genetically tractable vertebrate that serves as an indispensable animal model for studying human infectious diseases, particularly mycobacterial pathogenesis (172–177). *D. rerio* is the natural host for *M. marinum* (66, 178), and it stands out for its ability to recapitulate major pathological hallmarks of human TB, including the formation of well-organized, caseating granulomas (Figure 2) (66, 172–174, 176). The utility of the zebrafish model hinges on the distinction between its developmental stages concerning immune function: the early life stages (embryos and larvae) rely exclusively on the innate immune system, whereas the adult stage possesses both innate and adaptive immunity (172, 174, 179–181). Although the leopard frog, *Rana pipiens*, is also a natural host for *M. marinum*, the granulomas formed in this model are not caseating or necrotic (66). All these factors make the zebrafish-*M. marinum* model a great option to study early and chronic stages of Mtb. In the context of this review, zebrafish therefore extend the comparative spectrum from amoebae, nematodes, insects, and wax moth larvae to a vertebrate host, allowing conserved innate mechanisms to be examined within a tissue and granuloma architecture that closely parallels human TB.

### 5.1 Cellular immunity: macrophages, neutrophils, and mycobacterial exploitation

The early life stages of zebrafish possess a functional innate immune system, active from approximately 1 day post-fertilization, composed primarily of macrophages and neutrophils (11, 172, 179–183). Zebrafish macrophages are the first immune cells to arrive at the infection site and efficiently engulf the invading mycobacteria (179, 180, 182, 184). However, they possess a dichotomous role: while they restrict extracellular mycobacterial proliferation (172, 182), they also serve as replication niches and vehicles for dissemination, transporting mycobacteria to new tissues and thereby establishing secondary infection foci (11, 172, 174, 180, 182, 185, 186).

Pathogenic mycobacteria actively manipulate macrophage recruitment and polarization. The virulence lipid PGL attracts Ccr^2+^ monocytes that are permissive to bacterial growth (172, 174, 180), whereas PDIM masks TLR ligands, allowing the bacteria to evade detection by microbicidal, iNOS-expressing macrophages (66, 172, 174). These strategies enable the pathogen to skew early host responses toward a phenotype favorable for intracellular persistence.

Neutrophils, by contrast, exhibit distinct roles in the innate defense landscape. They are recruited to infection sites but rarely engulf extracellular *M. marinum* (180, 182). Instead, they perform efferocytosis—engulfing and clearing dead, infected macrophages—to prevent necrotic tissue damage and bacterial spread (180, 182, 187). A subset of neutrophils is capable of killing phagocytosed mycobacteria through the production of ROS and nitric oxide (NO) via NADPH oxidase activity (172, 174, 180, 182). Enhancing NO production by stabilizing Hypoxia-Inducible Factor 1-alpha (Hif-1) signaling in neutrophils has been shown to modulate susceptibility and reduce bacterial burden, highlighting the conserved role of hypoxia-responsive pathways in antimicrobial defense (172, 174, 186, 188, 189).

### 5.2 Granuloma biology: an innate immune structure and a pathogen-driven process

Optically transparent zebrafish larvae have been fundamental in revealing that granuloma formation is an active, dynamic process driven by both the host and the pathogen, initiated entirely within the context of innate immunity (172, 174, 180, 190). These structures arise when infected macrophages aggregate to form inflammatory lesions that serve both protective and pathogenic roles (11, 180, 182, 190).

The formation and expansion of early granulomas are actively driven by mycobacterial virulence (172, 174, 180, 186). The ESX-1 is essential for this process (172, 186), mediating programmed cell death (necrosis and pyroptosis) of infected macrophages (172, 174, 182, 186). Mutational studies in zebrafish have shown that early ESX-1 effectors, including those encoded in the RD1 locus and the accessory proteins EspK and EspL, are crucial for macrophage aggregation and early granuloma formation in *M. marinum*, although EspK appears dispensable for virulence in *M. bovis* and Mtb (66). This controlled cell death releases both bacteria and intracellular contents, attracting neighboring macrophages that engulf the debris and become secondarily infected. This process can also involve cell fusion between infected and uninfected macrophages (11, 172, 174, 180, 182, 186, 187, 191). The resulting cyclic recruitment and infection amplify bacterial dissemination and promote the development of multicellular granulomas.

Chemokine signaling plays a pivotal role in this process. The mycobacterial effector EsxA induces the expression of host matrix metalloproteinase 9 (MMP-9) in adjacent epithelial cells, which acts as a chemotactic cue for macrophage recruitment, thereby facilitating granuloma maturation and expansion (172, 185, 186, 190). This mechanism highlights how mycobacteria subvert host chemokine pathways to recruit permissive phagocytes and sustain infection.

### 5.3 Innate recognition, autophagy, and inflammatory balance

Zebrafish models have also elucidated the molecular and cellular pathways underlying innate immune recognition and effector responses. TLR signaling, mediated by the adaptor molecule MyD88, is essential for the induction of proinflammatory cytokines such as IL-1β and TNFα and provides protection during early pathogenesis (181, 183, 192). MyD88 deficiency leads to increased susceptibility and accelerated granuloma formation (174, 179, 181), underscoring the protective role of canonical pattern-recognition receptor pathways in early infection (181). Although TNF is necessary for macrophage microbicidal activity and granuloma stability, zebrafish studies reveal that early granuloma formation can proceed through TNF-independent mechanisms, with metalloproteinase 9 (MMP9) and Type 2 STAT6-driven pathways instead guiding epithelioid transformation and maturation, paralleling findings in mammalian models (66).

The JAK/STAT pathway in zebrafish is conserved and plays a pivotal role in the innate immune defense against mycobacteria, not only through classical cytokine signaling and immune cell recruitment but also by modulating host lipid metabolism within infected phagocytes (82, 193, 194). This dual function exemplifies how zebrafish integrate metabolic and immune responses to limit bacterial growth and inflammation.

Inflammasome signaling also plays a critical role in host defense. Activation of the Asc-dependent inflammasome promotes IL-1β secretion and pyroptotic cell death, thereby restricting intracellular bacterial growth and limiting granuloma expansion (180, 195). Conversely, regulatory NOD-like receptors such as Nlrc3-like act as negative modulators of inflammation. Loss of NLRC*3-like* enhances resistance by boosting inflammasome activation and proinflammatory cytokine production in infected macrophages (195). These findings illustrate the delicate equilibrium between inflammatory activation and resolution required to maintain effective yet non-pathogenic immunity.

Moreover, the autophagy machinery is a crucial host-protective defense mechanism against *M. marinum* (11, 172, 174, 180, 191, 196). Selective autophagy receptors (SLRs), such as Optn and p62, promote host resistance against *M. marinum* infection (196, 197). The DNA-damage-regulated autophagy modulator 1 (Dram1) links mycobacterial recognition via the TLR-MyD88 pathway to the autophagic defense mechanism, promoting the fusion of autophagosomes and lysosomes and enhancing host resistance (172, 174, 180, 196, 197). Nonetheless, pathogenic mycobacteria retain partial resistance to these defenses (197). *M. marinum* can inhibit phagosome-lysosome fusion and even survive within acidified compartments by expressing MarP, a virulence determinant required for acid tolerance and persistence (66, 198). These findings echo those from Dd and *Drosophila*, indicating that mycobacteria repeatedly target autophagy and membrane damage–repair pathways across host phyla, while hosts reuse a conserved set of stress- and danger-sensing modules to contain intracellular infection.

In addition, the zebrafish genome encodes orthologs of all key ESCRT components involved in endosomal sorting, membrane remodeling, and repair (199, 200). This conservation suggests that ESCRT-mediated membrane repair and vesicular trafficking play essential, though still underexplored, roles in the cellular response to mycobacterial infection, potentially analogous to their functions in mammalian macrophages.

A balanced inflammatory response is crucial for disease outcome. Both insufficient (hypo-inflammatory) and excessive (hyper-inflammatory) responses promote susceptibility (172, 174, 195). Overproduction of tumor necrosis factor (TNF) or loss of negative regulators such as Ptpn6 or Nlrc3-like precipitates necrotic macrophage death, fueling bacterial expansion (172, 174, 195). High TNF levels are particularly detrimental, as they trigger programmed necrotic cell death (necroptosis) in macrophages, leading to bacterial growth and spread (172, 174). Similarly, the CXCR3–CXCL11 axis, which orchestrates macrophage chemotaxis, is often exploited by mycobacteria to enhance dissemination; its disruption limits granuloma growth and bacterial spread (172, 174, 185).

**Figure 3:**
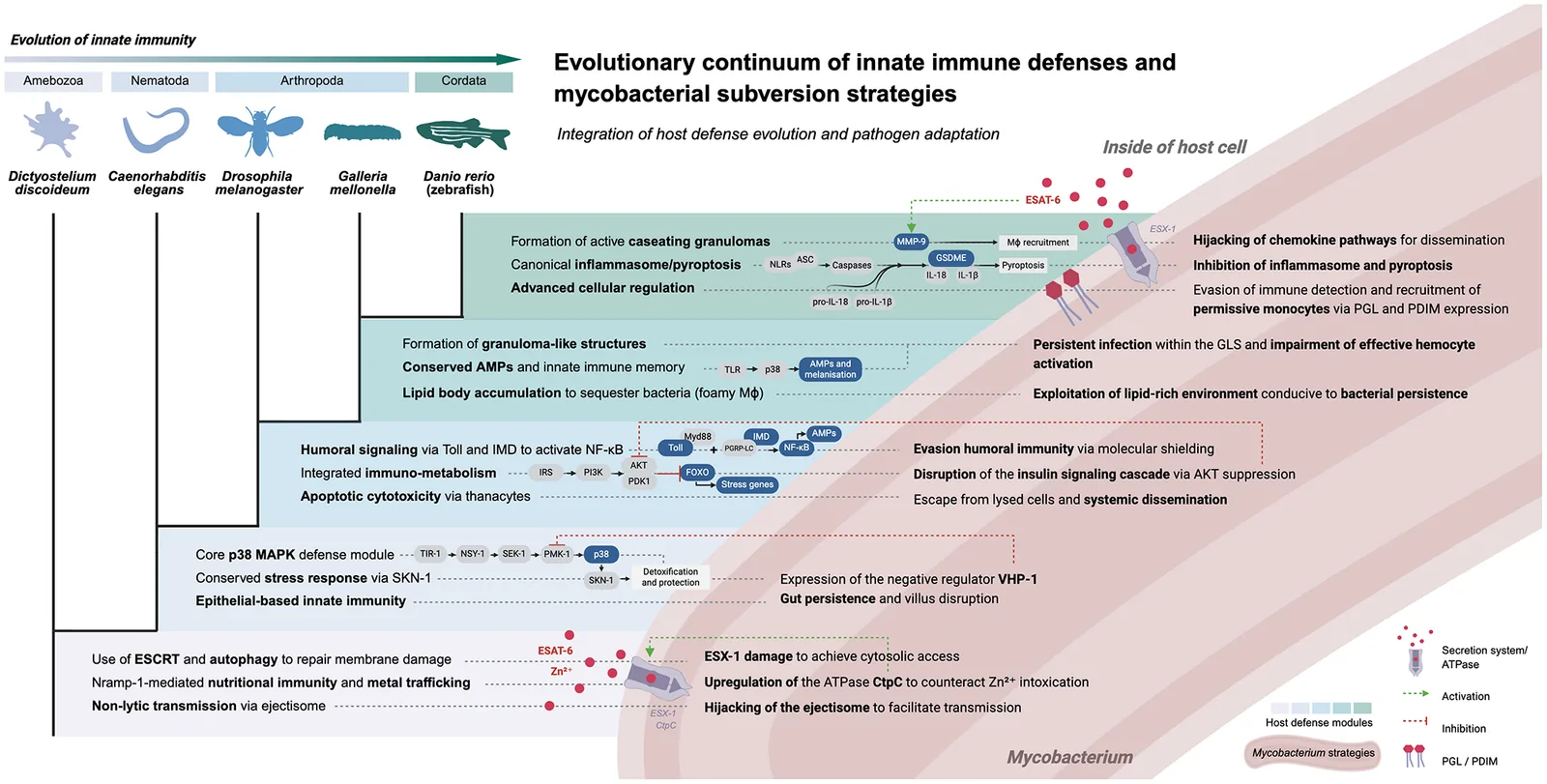
Evolutionary continuum of innate immune defenses and mycobacterial subversion strategies. The figure summarizes key innate immune pathways across reviewed host models and their corresponding mycobacterial countermeasures. It provides a comparative overview of notable pathways rather than an exhaustive catalog, emphasizing those mechanisms most relevant for understanding host–pathogen co-adaptation. From ancient cell-autonomous defenses in *Dictyostelium discoideum* (autophagy, ESCRT-mediated repair, Nramp1 metal deprivation) to epithelial immunity in *Caenorhabditis elegans* (PMK-1/p38 MAPK–SKN-1 axis), and cellular/humoral immunity in *Drosophila melanogaster* (Toll/IMD–NF-κB signaling, Akt–FOXO immunometabolic regulation), each model illustrates a distinct stage in the evolution of innate immunity. *Galleria mellonella* reflects advanced invertebrate immunity through granuloma-like structures, lipid-body accumulation, and innate immune memory. At the same time, *Danio rerio* represents the emergence of vertebrate features, including organized granulomas, inflammasome activation, and TNF-mediated regulation. In parallel, *Mycobacterium* species deploy conserved virulence strategies—such as ESX-1–mediated phagosomal damage, CtpC-dependent metal efflux, PDIM/PGL-driven immune evasion, and chemokine hijacking—underscoring the long-standing co-evolution between host defenses and mycobacterial persistence mechanisms.

In summary, the zebrafish larval model, relying entirely on innate immunity, has demonstrated that mycobacteria function as evolutionary drivers by subverting basic host innate mechanisms, exploiting macrophages for dissemination, hijacking chemokine signals for recruitment of permissive cells, actively driving pro-necrotic cell death to expand infection, and tolerating microbicidal strategies like phagolysosome acidification (172, 174, 180, 198). These host–pathogen interactions recapitulate the core immunopathological processes observed in vertebrate TB and underscore the zebrafish as a unique bridge between invertebrate models and mammalian immunity (177, 179, 192) (conceptual overview in Figure 3).

## 6 Discussion

Mycobacteria have served as powerful selective forces shaping the innate immune systems of various organisms throughout evolution. Studying these interactions across different host models reveals the ancient, conserved bases of immunity. The gradual adaptation of the *Mycobacterium* genus to intracellular life illustrates the spectrum from environmental opportunism to obligate pathogenicity (7, 12, 15, 201, 202). This process offers a framework for understanding bacterial evolution and provides insight into how ancient interactions with phagocytic hosts established conserved innate immune mechanisms. Despite extensive comparative work, the molecular basis of mycobacterial virulence remains poorly understood across host species, as many key determinants such as ESX-1 activity, lipid remodeling, and metabolic reprogramming show divergent outcomes in different models. A significant gap lies in defining which subversion mechanisms are truly conserved vs. host-specific, limiting our ability to translate findings from environmental and invertebrate systems to the pathogenic strategies of Mtb. Addressing these gaps requires integrative, multi-model frameworks that connect evolutionary conservation with clinical relevance.

The increasing use of non-mammalian hosts to study host-pathogen interactions offers practical advantages, such as ethical simplicity, experimental tractability, and phylogenetic diversity, making them essential complements to mammalian models (11, 47, 66, 149, 155, 160, 203). These systems connect environmental microbiology to human disease and provide new insights into immune resilience, tolerance, and trained immunity. For example, Dd shows that core, cell-autonomous defenses such as phagocytosis, nutritional immunity, autophagy, and ROS machinery, as well as ESCRT-mediated membrane repair mechanisms to counteract ESX-1 damage, are evolutionarily ancient, predating complex cytokine networks (42, 43, 49, 50, 52, 53, 63, 64, 74, 79, 183–185). Additionally, the p38 MAPK pathway is a key regulator of defense against mycobacteria in early metazoans, such as *C. elegans* and *Drosophila*, highlighting a conserved module linking stress responses and immunity (97, 99, 106, 143, 144).

However, while these non-mammalian hosts provide powerful insight into evolutionarily conserved innate defenses, they lack the full range of adaptive immune components. They cannot recapitulate T cell–mediated immunity, antibody responses, or the dynamic crosstalk between innate and adaptive pathways that critically shape mycobacterial disease. Thus, although these models are invaluable for dissecting ancient cell-autonomous defenses and core host–pathogen interactions, comprehensive investigation of adaptive immunity and clinically relevant TB pathogenesis requires the complementary use of evolutionarily closer systems, including murine models, other small mammals, and non-human primates.

In conclusion, the core architecture of innate immunity against mycobacterial infection evolved primarily through millions of years of coevolutionary struggle with mycobacteria in environmental and non-vertebrate hosts. The persistent dialogue between mycobacteria and their hosts continues to reveal the ancient origins and adaptive flexibility of immune defense. Therefore, comparative host models not only shed light on this shared evolutionary history but also help shape future strategies to fight mycobacterial disease within an evolutionary-informed framework.
