# Supplementary material for: Mycobacteria as evolutionary drivers of host innate immunity: insights from comparing experimental host models
Source: Front Tuberc. 2025 Dec 12;3:1735950. doi: 10.3389/ftubr.2025.1735950 (PMC13494977; doi:10.3389/ftubr.2025.1735950)
Supplement: Supplementary file 1 [file Data_Sheet_1.pdf]

Supplementary File 1 – Use of Generative AI Tools

Generative AI tools were used to assist in the language editing and structural refinement of the manuscript titled *"Mycobacteria as evolutionary drivers of host innate immunity: insights from comparing experimental host models."* All intellectual content, data interpretation, and scientific conclusions were generated and verified by the authors. The authors critically reviewed all AI-assisted text and assume full responsibility for the final manuscript.

Tools used:
ChatGPT (OpenAI, GPT-5 model).

Purpose of use:
To improve clarity, style, and consistency of scientific writing; to refine paragraph transitions; and to ensure terminological precision.

Example of initial prompt:
"Help me improve the clarity and flow of this section as part of a review discussing how mycobacteria have shaped the evolution of innate immunity across different experimental host models."

Example of final prompt:
"Refine the discussion paragraph to connect the identified gaps in understanding mycobacterial virulence with a translational framework integrating comparative models and clinically relevant surrogate endpoints."
